# Supplementary material for: Decentralized governance may lead to higher infection levels and sub-optimal releases of quarantines amid the COVID-19 pandemic
Source: PLoS One. 2022 Dec 15;17(12):e0279106. doi: 10.1371/journal.pone.0279106 (PMC9754229; doi:10.1371/journal.pone.0279106)
Supplement: Attachment — Submitted filename: Lampert_ResponseReviewers.pdf [file pone.0279106.s001.pdf]

Reviewer 1

I sincerely thank the Reviewer for carefully reading the manuscript and for the helpful comments. Below please find the Reviewer's comment (black fonts) followed by my response (blue & bold fonts).

This manuscript studies a decentralized decision-making problem, in which governors of two connected countries decide when to release restricted quarantine policies for their own populations. Residents of each country also travel to the other at some fixed rates, when the quarantine policy is lifted. The authors find that, in general, decentralized policies (under a Nash equilibrium) induce more infections on average than the optimal centralized decision-making policy.

I find the topic of this study to be interesting and important. I believe the analysis and contributions can be quite valuable. The stated conclusions are clearly laid out.

I thank the Reviewer for finding the study interesting and important.

My concerns are in regards to the methods, specifically the optimal control formulation. There were a few things that were not clear to me, and I thought some parts could be improved.

The Reviewer asks that I improve the clarity of the Methods section. In the revised version, I combined the "model" and the "methods" sections, and I describe the methods in more detail. In particular, I clarify the related issues raised by the Reviewer below.

- What is the space of control strategies for each player? Can they implement/lift restrictive quarantines multiple times throughout the dynamics, or is it only once? It would help to formally define this.

Each player/country dictates the time when it switches from a restrictive to a non-restrictive quarantine (T_i for country i). In the revised manuscript, I explain that "The government in each country dictates the time T_i at which it switches from a non-restrictive quarantine to a restrictive one" (lines 80-81).

Also, we consider a single cycle during which, initially, the infection level I_i declines until $t = T_i$, then I_i increases until $I_i = I_{\max}$, and then the government switches back to a restrictive quarantine and I_i declines again. In the revised manuscript, I explain that we "consider a cycle during which, initially, a restrictive quarantine is administered until $t = T_i$ (stage 1); afterward, a non-restrictive quarantine is administered until $I = I_{\max}$ (stage 2); and finally, the quarantine becomes restrictive again until the infection level returns to its original level $I_i(0)$ (stage 3)" (lines 86-88).

- The application of Pontryagin's maximum principle is not clearly detailed. If the space of control strategies is itself a timing decision, then the optimal response will be a timing decision anyway. I believe what can formally be shown here is that the optimal policy for a player is a bang-bang controller (i.e. zero-one policy), even when the space of control strategies are continuous (i.e. can select in the interval [0,1]).

The Reviewer is correct that the strategy of the government is regarding a single timing, and therefore, we do not need to use Pontryagin's maximum principle. The idea of the comment about Pontryagin's maximum principle in the original manuscript has been, as the Reviewer suggested, to explain why we consider a single switch and not, say, multiple switches during a cycle. However, based on this comment, I believe that this only adds confusion in the present context, and therefore, I decided to remove this comment.

- The objective function in equation (5) appears to be loosely defined. Why is it certain that the infectious level will return to its original level I_i(0)? This may follow from the setup, but please do explain this.

The objective function is defined as maximizing the relative amount of time that the country administers a non-restrictive quarantine during a full cycle. The full cycle is defined from some initial time ($t = 0$) until the time when I_i goes back to its initial level. In the revised manuscript, I clarify that "the objective of each government is to choose T_i that maximizes the portion of time during which a non-restrictive quarantine is administered, under the assumption that the quarantine becomes restrictive when I_i approaches $I_{\max}$. Specifically, consider a cycle during which, initially, a restrictive quarantine is administered until $t = T_i$ (stage 1); afterward, a non-restrictive quarantine is administered until $I = I_{\max}$ (stage 2); and finally, the quarantine becomes restrictive again until the infection level returns to its original level $I_i(0)$ (stage 3). In turn, denote T_i^{non} as the total time during the cycle when a non-restrictive quarantine is administered in country i (namely, ΔT_i^{non} is the duration of stage 2). Denote ΔT_i^{res} as the total time during the cycle when a restrictive quarantine is administered in country i (namely, ΔT_i^{non} is the duration of stages 1 and 3 combined). Using these notations, we define the utility of country i as $u_i(T_1, T_2) = \frac{\Delta T_i^{\text{non}}}{\Delta T_i^{\text{non}} + \Delta T_i^{\text{res}}}$" (lines 83-93 and Eq. 4).

- Is it possible to establish some properties regarding the best-response or Nash equilibrium strategies? For example, is it always ensured that one exists? From the simulations, it appears that one does exist, and that it is unique. Is it possible for there to be multiple equilibria?

The Reviewer asks whether a Nash equilibrium always exists in our model, and whether the Nash equilibrium is always unique. In all of my simulations, the answer is yes – I always obtained a single, unique Nash equilibrium, as shown in Fig. 2A,C,E (the red and the blue lines intersect exactly once in each example). Nevertheless, this of course does not guarantee that this is always the case, and perhaps there could be parameter values for

which a Nash equilibrium does not exist or is not unique. To clarify this, I wrote that "in general, a Nash equilibrium is not necessarily unique: there could be cases in which multiple Nash equilibria exist, and cases in which no Nash equilibrium exists [35]" (lines 99-101). I also comment in the legend of Fig. 2 that "a unique Nash equilibrium exists for each of the demonstrated cases" (lines 200-201).

- The "brute force" numerical methods appear to work for the selected parameters, but may not generalize or scale to other scenarios. They are also not standard in optimal control or differential game studies. I would recommend using more specialized optimal control algorithms, such as forward-backward sweep methods, shooting methods, and gradient descent. See for example,

M. McAsey, L. Mou, and W. Han, "Convergence of the forward backward sweep method in optimal control," Computational Optimization and Applications, vol. 53, no. 1, pp. 207–226, 2012.

The Reviewer notes that we could use other numerical methods to find the Nash equilibrium. While this is correct, note that the method that we used is also a standard method for finding the Nash equilibrium. In particular, it is a straight-forward generalization of the standard method for finding the Nash equilibrium in discrete games into games with continuous strategies. There are, of course, numerous alternative methods that could work as well, but I see no reason to redo the analysis because our method works perfectly fine and finds the exact solution. I revised the "numerical methods" sub-section to describe the algorithm more clearly.

I sincerely thank the Reviewer again for the valuable comments that helped to improve the manuscript.

Reviewer 2

I sincerely thank the Reviewer for carefully reading the manuscript and for the helpful comments. Below please find the Reviewer's comment (black fonts) followed by my response (blue & bold fonts).

The manuscript is based on mobility restrictions set by the governments within countries/states with the aim of maximizing the non-restrictive quarantine time for the citizens. In view of this, I would like the authors to address the following comments:

MINOR COMMENTS

There are quite a number of grammatical errors in the manuscripts which in my opinion, will give the reader a hard time to understand. For example, the following lines should be rephrased:

line 42 - a mistake I guess; "COPVID-19 outbreaks"

Fixed (line 44)

line 53-53 - the sentences are unclear; e.g., bracket at the beginning of a sentence

We rephrased the paragraph for clarity (lines 56-61).

line 77 - "...between the countries..." should be "...between the countries/states..." since CASE 3 described in Figure 1C considered only a state in a country

Fixed (line 144)

line 87-88 - the sentences are unclear

I rephrased the sentence for clarity. I also moved this sentence to the legend of Fig. 2, where its context is clearer (lines 207-210).

line 161-162 - the sentences are unclear; e.g., "... are in travel..."

I rephrased the sentence: "we denote μ_{ij} as the portion of county i's residents that travel to country $j \neq i$" (line 69).

line 169 - a mistake I guess; "is s/he" --> you meant "...if he/she...?"

Fixed (line 71)

line 52 & 150 - is a negative sign preceding r0 or a hyphen? If it is a negative sign, I suggest it is put in a bracket. On the other hand, if it is a hyphen, it should be replaced with a comma so as not to confuse the reader.

These are minus signs. In the revised manuscript, I rewrote the sentences to avoid confusion, e.g.: "when the quarantine in country i is restrictive, I_i decreases exponentially (increases at a negative rate $r_0 < 0$)…" (lines 56-58).

MAJOR COMMENTS

1. Firstly, a table describing all notations of sets, variables and parameters should be included in the manuscript; with a column showing the citations of any notation drawn from the literature.

Per the Reviewer's request, I added Table 1: list of notations to the manuscript (page 9). Note that the citations of the literature appear throughout the text, and the estimated values appear in the "parameterization" sub-section.

2. "Option 1" defined in the Methods is counter-intuitive. I believe line 153 should be "...non-restrictive to restrictive...". Otherwise, Equation 1 will not hold.

The Reviewer is right that the correct form is "from non-restrictive to restrictive" instead of the other way around. I fixed it in the revised manuscript. In addition, I rephrased the entire paragraph for clarity (lines 56-61).

3. Equation 4 should be explicitly explained. Additionally, when you consider what is written in line 171, Equation 4 appears to be incorrect as it will be impossible to arrive at Equation 3.

Eq. 4 in the original version of the manuscript was presented only as an ansatz to show the differences from Eq. 3. I did not use this equation to generate the results (Figs. 2 and 3); rather, I used Eqs. 1-3. Therefore, in the revised manuscript, I removed Eq. 4 to avoid confusion.

4. It is not clear how "Option 1", "Option 2", and "Option 3" described in the Methods section (line 144-175) are linked with the cases discussed in Figure 1 and how it was explained in the results. That is, what is the role of "Option 1/Option 2/Option 3" in Case 1/Case 2/ Case 3? Are all options considered in each of the cases or some of the options are considered? These are unclear and needs to be explicitly discussed.

The term "options" that I used in the original version of the manuscript described the different situations that each country could experience (being under a restrictive quarantine; being under a non-restrictive quarantine while the other country is under a restrictive quarantine; both countries are under a restrictive quarantine). These "options"

are not related to the "cases" demonstrated in Fig. 1. Rather, each of the three "options" occurs in all three cases.

In the revised manuscript, I removed the poorly-chosen word "option" to avoid confusion. Instead, I rephrased the entire section and combined what had previously appeared in Methods into the Model. In the revised manuscript, the dynamics of the population are detailed in their entirety in lines 51-79. In particular, the description details the dynamics under restrictive quarantine (lines 56-63 and Eq. 1); the dynamics under a non-restrictive quarantine when the other country is still under a restrictive one (lines 63-67 and Eq. 2); and the dynamics when both countries are under a non-restrictive quarantine (lines 68-79 and Eq. 3).

5. The methods used in obtaining the "socially optimal solution" should be explained in details and discussed separately under the Method sections and also show this explicitly in Figure 2 and Figure 3 since the results described how the Nash equilibrium was compared to the socially optimal solution.

In the revised manuscript, I extended and clarified the explanation of how we found the socially optimal solution, as requested by the Reviewer. First, I explain that "we find the socially optimal solution, T_1^{opt} and T_2^{opt}, which maximizes the utility of the entire society in both countries combined (i.e., the combination $T_1 = T_1^{\mathrm{opt}}$ and $T_2 = T_2^{\mathrm{opt}}$ maximizes $N_1 u_1(T_1, T_2) + N_2 u_2(T_1, T_2)$)" (lines 94-96). I also clarified how we define $u_1(T_1, T_2)$ in lines 80-99 and Eq. 4. Furthermore, I revised the "numerical methods" sub-section for clarity and I explain the algorithm for calculating the socially optimal solution (lines 106-117).

Regarding the request to demonstrate the optimal solution in the figures, note first that the optimal solutions are already demonstrated and compared to the Nash equilibria in Fig 2, panels B, D, and F. In the revised manuscript, I added a purple dot to each of the panels A, C, and E of Fig. 2, indicating the T_1 and T_2 values of the socially optimal solution. I also changed the notations in these panels to ones that are more clearly defined in the text.

In turn, note that Fig. 3 only shows the reduction in the country's utility, comparing between the case in which the countries adopt the Nash equilibrium and the case in which they adopt the optimal solution. Showing either the Nash equilibrium or the optimal solution on that graph would be meaningless. I revised the legend of Fig. 3 for clarity.

I sincerely thank the Reviewer again for the valuable comments that helped to improve the manuscript.
