# Supplementary material for: Decentralized governance may lead to higher infection levels and sub-optimal releases of quarantines amid the COVID-19 pandemic
Source: PLoS One. 2022 Dec 15;17(12):e0279106. doi: 10.1371/journal.pone.0279106 (PMC9754229; doi:10.1371/journal.pone.0279106)
Supplement: Attachment — Submitted filename: ResponseReviewers.pdf [file pone.0279106.s002.pdf]

Reviewer 3

I sincerely thank the Reviewer for carefully reading the manuscript and for the helpful comments. Below please find the Reviewer's comment (black fonts), followed by our response (blue & bold fonts). Changes from the previous version are also highlighted in yellow in the marked manuscript.

The paper analyzes the role of decentralized control of epidemic spreading over sub-populations through lock-down interventions. Through constructing the cost function as a function of the intervention, the paper studies the optimal- and sub-optimal intervention strategies. Analysis of a Nash equilibrium of the model is given. I have the following major concerns about the paper.

1) My biggest concern is that the paper studies an epidemic spreading over a two-country spreading model. The results are developed on the two-country spreading model. In real-world scenarios, we might need a generalized multi-population spreading model. How can the results developed in this work be applied to a more generalized multi-population spreading model, in terms of theoretical analysis of the equilibrium, comparison of the optimal intervention period, and the numerical analysis of the solution?

This manuscript focuses on the case of two countries or states, as the Reviewer correctly points. At the same time, some of the results are more general and can be generalized to multiple countries. For example, the fact that the quarantine management by multiple countries is sub-optimal is likely to hold in cases of more than two countries. Furthermore, based on previous studies in game theory, it is reasonable to hypothesize that increasing the number of countries will make the Nash equilibrium solution even less efficient.

Of course, future studies will need to examine how exactly the results scale with the number of countries. Note, however, that the model itself is already written in a form that allows more than two countries. Therefore, future studies can analyze the same model with more countries to examine the effect of increasing the number of countries.

In the revised manuscript, we acknowledge this in the Discussion when we discuss the study's limitations: "… we have made numerous simplifying assumptions in our model, which suggests that there are various future directions for examining the consequences of relaxing these assumptions. First, we consider only two countries or states, whereas in reality, these are transmissions between multiple countries and states" (lines 177-180).

2) Starting from line 29, the paper claims that "each government might ignore the cost borne by citizens of other countries due to international and interstate travel". In reality, e.g., during the COVID-19 pandemic, it is more common for a country to only considers the impact/cost of the intervention within the country itself, instead of the what the intervention will bring to other countries. The paper should give some solid examples to show the reason why a country should consider the impact of the intervention on other countries.

In the paper, we assume that each country/government cares only for the welfare of its own citizens. Using this assumption, we calculate the Nash equilibrium. In turn, we compare this solution to a hypothetical scenario in which a single government makes the decision for both countries/states, which we refer to as the optimal solution. Indeed, as the Reviewer suggests, we expect each governor to maximize the welfare in her/his own country, and therefore, the Nash equilibrium outcome is the expected outcome.

I understand, however, where the confusion came from. In the previous version, I wrote that "the objective of each government is to choose T_i that maximizes the portion of time during which a non-restrictive quarantine is administered." In the revised manuscript, I clarify that the objective of the government in a given country is to maximize the portion of time during which a non-restrictive quarantine is administered *in its own country*: "the objective of each government is to choose T_i that maximizes the portion of time during which a non-restrictive quarantine is administered in its own country, country i..." (lines 85-87).

3) The paper gives three assumptions from line 38 to line 41. All assumptions assume both countries have around the same population, which are strong assumptions in real world. Is there a way to relax the population assumptions? In addition, under the assumption that both countries have around the same population, is it necessary that the cost function given in line 96, i.e., 11(1, 2) + 22(1, 2)) contains N1 and N2?

We do not assume that all countries have the same population size. Rather, we examine three cases/scenarios. In cases number 1 and 2, we indeed assume that the population size in both countries is the same; however, in case no. 3, we examine the scenario in which the countries have different population sizes.

To clarify this issue, I rephrased lines 37-41, and I explain that "in case 1, we consider the scenario in which ...;" "in case 2, we consider the scenario in which ..." I believe that this clarifies what we consider in each case separately.

4) In line 60, the paper assumes that the infected population of each country cannot be lower than a threshold. The assumption is in general good. However, extremely strict lock-down can eradicate the epidemic completely, e.g., China did it during 2021. It might be better to further clarity the definition of restrictive quarantine and non-restrictive quarantine in this paper. If the restrictive quarantine is as restrictive as China did before, it is possible that the infected population can reach zero for a while.

I thank the Reviewer for this comment. Indeed, the "restrictive" quarantine that we consider is one like in the US or Europe, which has been useful in reducing the infection level but not in eliminating it like in China. In the revised manuscript, I explain that "we consider a relatively restrictive quarantine, but one that still allows for some local mobility and does not eliminate the disease entirely" (lines 63-64).

5) Is equation (3) a popular way of modeling epidemic transmission? If so, please add a couple of reference. From my own understanding, the transmission and infection are usually model through the Law of mass action, given by nonlinear dynamics. However, equation (3) uses a group of linear dynamics to capture the change of the infected population, which may not be well-accepted in the epidemiology and disease spreading area.

There are two approaches for disease modeling when two countries/states/cities are coupled. One is the Eulerian approach, assuming that transmissions between the countries are due to people migrating from one country to another. The second approach is the Lagrangian approach, assuming that transmissions occur when residents of one country visit other countries, but these visitors remain residents of their home country and ultimately return.

In the manuscript, I explain that "We assume that a person that travels from country 1 to country 2 is still a resident of country 1 who will return to country 1. Therefore, if this traveler gets infected, this increases I_1 and not I_2, even if the transmission has occurred in country 2 [32-35] (Lagrangian approach)" (lines 72-74). In particular, this sentence has been revised for clarity and I provide the new references 34 and 35.

In turn, the explanation of why the model is quasi-linear is given at the beginning of the Model section in lines 45-50: "we assume that the number of infected people in each country is very small compared to the country's total population size. Accordingly, we consider only the dynamics of the infection level in each country, I_i, defined as the portion of individuals that are infected in country i. Namely, in contrast to traditional SIR models [31], here we consider shorter timescales during which the number of susceptible individuals is constant, which is in line with the COVID-19 data…"

6) The descriptions of strictive and non-strictive quarantine from 89 to 92 do not match the introductions of these three stages from line 82 to line 88. It is confusing that the definition of Stage 1 means the beginning of a stage or the end of a stage.

In lines 89-92, I simply "split" the period during which the restrictive quarantine is administered, for the purpose of explaining the technical definition of a cycle: "stage 1" from the beginning until $I_i = I_{\min}$, and "stage 3" from the time when I_i becomes $I_{\max}$ until I_i returns back to its initial value $I_i(0)$. There is no inconsistency in the definitions. To further clarify this, I explain in the revised manuscript that "In particular, if initially $I_i(0) = I_{\max}$, then the duration of stage 3 is T_{delay}" (lines 91-92).

7) In the section "Optimal solution and Nash equilibrium", the paper gives the socially optimal solution. Can the paper give more intuitive explanations about the solutions in real world implementation? The paper also mentioned that there could be cases in which multiple Nash equilibria exist, and cases in which no Nash equilibrium exists. Can the paper give more detailed information on when and how these cases appear under the current setting?

The optimal solution is used in the paper as an "ideal" situation that maximizes the welfare of society as a whole. This is compared to the "actual" expected solution of self-interested

governments, given by the Nash equilibrium. In turn, for another interpretation of the optimal solution, one could expect the optimal solution to represent a case in which a single government makes the decision regarding the quarantine policy in both states. In the revised manuscript, I added the explanation that “This solution represents the best quarantine policy in a case where a single governor makes the decision for both states” (lines 99-101). I also mention in the introduction that “we examine how the strategies of different governments differ from the socially optimal strategy of a hypothetical centralized government that aims to maximize the welfare of all the citizens in all the countries” (lines 34-36).

Regarding the Nash equilibrium, my results show that a unique Nash equilibrium exists in all the cases that I have examined: “a unique Nash equilibrium exists for each of the three demonstrated cases” (lines 205-206). However, one of the reviewers of the previous version has asked me to add a comment acknowledging that, in general (in some other games), a Nash equilibrium does not have to be unique. I revised the sentence for clarity and I added that a Nash equilibrium is not necessarily unique “in some games” (line 104). This emphasizes that this is a general comment and that I do not argue that this is the case in the game studied in this manuscript.

8) In the numerical simulation, how scalable the proposed method can be? The paper considers a two-country case. What if we need to consider twenty or even more countries? Can the numerical method be implemented to large-scale networked spreading models?

The numerical algorithm can indeed be generalized to cases that include more than two players. However, I had no reason to implement the algorithm beyond what I needed for this study. Future studies by myself and/or others can use an extended version of the algorithm. To clarify this in the revised manuscript, I added that “We implemented here the algorithm for the case of two countries” (lines 111-112).

I thank the Reviewer again for the valuable comments that helped to improve the manuscript.
